# Comment on “Empirical comparisons of heterogeneity magnitudes of the risk difference, relative risk, and odds ratio”

**DOI:** 10.1186/s13643-025-02758-7

**Published:** 2025-03-27

**Authors:** Hirotaka Mori

**Affiliations:** Department of Biostatistics, Graduate School of Medicine, Hokkaido University, Kita-Ku, Sapporo, Hokkaido N15 W7 Japan

I am writing regarding the article “Empirical comparisons of heterogeneity magnitudes of the risk difference, relative risk, and odds ratio” by Zhao et al. (Systematic Reviews, 2022;11:26). This study makes a valuable contribution to the field of meta-analysis by providing empirical evidence about the comparative behavior of different effect measures. The finding that approximately half of the analyzed meta-analyses show low heterogeneity is particularly important for researchers conducting meta-analyses, as it helps inform methodological choices and interpretation of results.

However, I identified some numerical inconsistencies that I believe warrant clarification to ensure the accurate interpretation of these important results.

First, regarding Table S2 in the Supplemental File, I noticed that the sum of meta-analyses across the three categories ($I^2=0\%,\;0\%<I^2\;\leq1\%$, and $I^2>1\%$) does not equal the total number of analyzed meta-analyses (64,929) for any of the models or effect measures. This discrepancy may affect the interpretation of the proportion of low-heterogeneity meta-analyses in practice.

Second, I carefully reconstructed the histogram data for meta-analyses with ${I}^{2}>1\%$ shown in Fig. 2A using the Degitizit software. For the relative risk measure, I found that the number of meta-analyses was 21,776 under the REML method and 21,875 under the DL method. However, Table S2 in the Supplemental File reports different numbers: 37,607 for REML and 37,020 for DL. Understanding the source of this difference would be valuable for researchers who wish to build upon these findings.

I would appreciate if the authors could help clarify these numerical discrepancies. Their clarification would strengthen the utility of these important findings for future research and meta-analytic practice. The resolution of these questions would be particularly valuable for those who are conducting research on heterogeneity patterns in meta-analyses.

I thank the authors for their significant contribution to our understanding of heterogeneity measures, and I look forward to their response, which will help ensure the accurate application of their findings in future research.

